# The association of pregnancy complications/risk factors with the development of cancer in women: an umbrella review

**DOI:** 10.1186/s12916-026-04844-6

**Published:** 2026-04-13

**Authors:** Jemma Healey, Megha Singh, Clare Richards, Rachel Plachcinski, Ngawai Moss, Leila Ghalichi, Siang Ing Lee, Stephanie Hanley, Zoe Vowles, Francesca L. Crowe, Krishnarajah Nirantharakumar, Mairead Black

**Affiliations:** 1 University of Aberdeen, Aberdeen, Scotland, UK; 2 NHS Grampian, Aberdeen, Scotland, UK; 3 Institute of Applied Health Sciences, University of Birmingham, Birmingham, UK; 4 Patient and Public Representative, London, UK; 5 Guy’s and St., Thomas’ NHS Foundation Trust , London, UK; 6 School of Life Course and Population Sciences, King’s College London, London, UK; 7 Aberdeen Centre for Women’s Health Research, School of Medicine, Medical Science and Nutrition, University of Aberdeen, Aberdeen, UK

**Keywords:** Pregnancy complication, Gestational diabetes, Cancer, Ovarian cancer, Pre-eclampsia

## Abstract

**Background:**

Among women in the UK, over 186,000 new cancer diagnoses and around 78,000 cancer deaths occurred annually from 2017 to 2019. Evidence suggests that pregnancy complications are linked to mortality and morbidity risks in later life. This umbrella review aims to assess the association between pregnancy complications and cancer risk. It forms part of a series of studies exploring associations between pregnancy complications and long-term health conditions.

**Methods:**

MEDLINE, Embase, and Cochrane databases were searched from inception to April 2024. Key search terms encompassed ‘cancer’ and ‘pregnancy complications’ or ‘pregnancy risk factors’. Screening data extraction and quality appraisal (AMSTAR 2) were completed by two independent reviewers. Data were synthesised narratively and quantitatively. Relative risks (RR)/odds ratio (OR)/hazard ratios (HR) with 95% confidence intervals were reported.

**Results:**

Of the 25 reviews assessed for methodological quality, 2 were rated high, 12 moderate, 9 low, and 2 critically low. After excluding 10 overlapping reviews and 2 critically low reviews, 13 reviews included reviews consisted of 170 primary studies. Associations between 7 pregnancy complications and 17 cancers are reported. Women with molar pregnancy had four-fold higher risk of developing gestational trophoblastic neoplasia [OR 4.72 (1.81–12.32)]. Miscarriage was associated with thyroid cancer [OR 1.29 (95% CI 1.15–1.44)], but not with breast or ovarian cancer. Pre-eclampsia was associated with a reduced risk of breast cancer [RR 0.89 (0.83–0.95)] and an almost twofold higher risk of ovarian cancer [RR 1.82 (1.16–2.85)]. Gestational diabetes mellitus was associated with a higher risk of thyroid cancer [RR 1.28 (1.16–1.42)], stomach cancer [RR 1.43 (1.02–2.00)], liver cancer [RR 1.27 (1.03–1.55)], and blood cancer [RR 1.48 (1.04–2.09)] but was not associated with other cancers studied. Preterm birth showed a very small association with breast cancer risk (OR 1.03, 95% CI 1.00–1.07). There was no significant association between caesarean section and cervical cancer or multiple births and breast cancer.

**Conclusions:**

Some pregnancy complications were associated with selected cancer outcomes, although the evidence was heterogeneous and limited by potential bias, confounding, and inconsistent review quality.

**Supplementary Information:**

The online version contains supplementary material available at 10.1186/s12916-026-04844-6.

## Background

Cancer is the most common cause of death for women aged 35 to 79 in England and Wales [1]. Among women in the UK, over 186,000 new cancer diagnoses and around 78,000 cancer deaths occurred each year from 2017 to 2019 [2]. In the past 10 years, cancer rates in women in the UK have risen by 5%, with multifactorial causes suspected [3].

Pregnancy complications have been linked to increased risk of subsequent mortality and morbidity, particularly for metabolic and vascular disorders. For example, women who experience pre-eclampsia, gestational diabetes mellitus (GDM), or foetal growth impairment are at greater risk of developing cardiovascular disease and metabolic conditions [4]. Consequently, whether pregnancy caused the long-term conditions or if underlying susceptibility to health conditions was expressed during pregnancy is hotly debated [5].

Pregnancy also appears to protect against the development of some cancers [6], including a 30–60% reduced risk of ovarian cancer in parous women. This may be important as fertility rates continue to fall in the UK [7]. Younger age at first birth, pre-eclampsia, and breastfeeding are associated with a lower risk of breast cancer.

Pregnancy complications linked to increased risk of cancer include gestational diabetes mellitus (GDM), which is associated with other complications such as caesarean birth and has been linked to increased risk of haematological, ovarian, uterine, and breast cancers [8, 9]. Fuchs et al. hypothesised that GDM may increase ovarian, uterine, and breast cancers due to hyperinsulinaemia and increased insulin-like growth factor 1 (IGF-1) cascade, prompting growth of cancer cells. Alternatively, oestrogen production by adipose tissue may be involved as GDM is more common with obesity. The link between GDM and cancer may have significant impact as GDM affects 2–9% of pregnancies in Europe, Australia, and the USA. Synthesised data on the relationship between several pregnancy complications and cancer risk come from differing populations and, in some cases, data are conflicting with elements of bias and unmeasured confounding present.

Routine antenatal and postnatal care provides a unique opportunity for women who ordinarily do not have regular health monitoring to have access to screening and disease prevention strategies. This approach has been utilised to reduce future risk of morbidity for both GDM and pre-eclampsia. By making primary care providers aware of the conditions and associated risks [10], it is intended to prompt screening and prevention programmes [10].

This study aims to assess the associations between pregnancy complications (20) and risk of cancer as part of a wider project exploring the association between pregnancy complications and long-term health conditions. An umbrella review, which is a review of existing systematic reviews and meta-analyses, was conducted.

## Methods

This umbrella review aims to summarise the evidence available in the form of systematic reviews assessing the association between pregnancy complications and development of cancers in women. It has been conducted in accordance with Joanna Briggs Institute (JBI) umbrella review methodology [11]. The Preferred Reporting Items for Overviews of Reviews (PRIOR) checklist was used to report the review (Additional file 1: Table 1) [12]. The published protocol has been registered on PROSPERO (registration number CRD4202233499) [13].

### Population, exposure, and outcomes

The target population was women who had pregnancy complications or risk factors without any age restriction. The exposures (pregnancy complications/risk factors) were identified after a scoping review [13] followed by consensus with an expert panel (clinicians, obstetricians, epidemiologists, and patient and public representatives—MuM-PreDiCT) to determine the final list of exposures to be searched. The list of exposures is reported in Table 1.

**Table 1:** List of exposures

1. Hyperemesis gravidarum
2. Ectopic pregnancy
3. Pregnancy loss—miscarriage/recurrent miscarriage/spontaneous pregnancy loss
4. Hypertensive disorders of pregnancy—gestational hypertension, pre-eclampsia—early or late onset, recurrent pre-eclampsia, eclampsia, haemolysis, elevated liver enzymes and low platelets (HELLP) syndrome
5. Placental disorders: placenta previa, placental abruption, placenta accreta, placenta percreta
6. Gestational diabetes mellitus (GDM)
7. Molar pregnancy/choriocarcinoma
8. Multiple pregnancy/twin pregnancies/multiple gestation
9. Obstetric haemorrhage (post-partum)
10. Preterm birth/recurrent preterm birth
11. Mode of birth: caesarean, instrumental
12. Low birth weight
13. Small for gestational age
14. Intrauterine growth retardation/intrauterine growth restriction
15. Perineal trauma: third degree and fourth degree tear
16. Obstetric cholestasis
17. Pelvic girdle pain
18. Stillbirth
19. Postpartum depression
20. Puerperal psychosis

Outcomes were the development of cancers. The list of included cancers was agreed upon following a scoping search, and a discussion with the expert panel, with considerations given to cancers common in women of reproductive age and later life. The cancers included were brain and other central nervous system and intracranial tumours, cervical, colorectal, leukaemia, liver, lung, melanoma, non-Hodgkin lymphoma, oesophageal, ovarian, pancreatic, renal, skin, thyroid, and uterine.

### Study design, selection, and data extraction

Systematic reviews with or without meta-analyses were included. Eligible studies assessed the association between pregnancy complications/risk factors and future risk of cancer. A review was considered eligible if it described a systematic methodological approach and could demonstrate it had attempted to identify and synthesise the available literature required to address the research question. Scoping reviews, narrative reviews, commentaries, and guidelines were excluded.

The search was conducted in MEDLINE, Embase, and Cochrane library of systematic reviews from inception until April 2024. No restriction of language was employed. The key search terms captured the concept of pregnancy complications, carcinoma, and cancer. The results were limited using a search filter to systematic reviews and meta-analysis. The reference lists of included reviews were searched. The full search strategy for MEDLINE is provided in Additional file 1: Table 1. After removing duplicate studies, two independent reviewers conducted the title and abstract screening. Two reviewers (JH and CR) independently screened the full papers. A third reviewer (MB) was consulted in case of discrepancy. One review was published in Iranian. A fellow researcher (LG) with expertise in the Iranian language was consulted, translated the review, and performed the data extraction and quality assessment.

### Data extraction

Two independent reviewers (JH and CR) extracted the data from the reviews and in case of discrepancy, a third reviewer (MB) was consulted. Data was extracted under the following headings: aim of the review; database searched; search period; exposures; comparator; outcomes; study design(s); definition of exposure; definition of outcome; data synthesis method; quality assessment tool; quality of the included primary studies as assessed by review authors; characteristics of the included reviews; outcome measures; and conclusion of the review. The authors of the reviews were contacted where further information was required.

### Quality assessment

The quality of each eligible review was appraised using A Measurement Tool to Assess systematic Reviews 2 (AMSTAR 2) [14]. Two reviewers (JH and CR) independently conducted the quality assessment, and the third reviewer was consulted in case of discrepancy. The AMSTAR 2 tool contains 16 domains, and one point is assigned to each domain if fulfilled. The review is categorised as high, moderate, low, or critically low quality depending upon the domains fulfilled. The domains considered critical in the checklist were registration of the protocol before starting the review; conduct of an adequate search of the literature; providing justification for the exclusion of individual studies; satisfactory assessment of risk of bias in the studies included in the reviews; use of appropriate statistical methods in performing a meta-analysis; and accounting for risk of bias when interpreting the results. If any of these critical domains were not fulfilled, then the review was rated as low quality. The reviews rated as critically low quality were excluded. Details of quality assessment are presented in Additional file 1: Table 3.

### Overlap of reviews and outdated reviews

Where two or more reviews investigated the same research question, it is possible that the same primary studies may have been included and therefore the reviews may overlap. In such instances, a citation matrix of the primary studies was plotted, and the degree of overlap was assessed through calculating the corrected covered area (CCA) [15, 16]. A citation matrix was constructed indicating primary studies in rows and systematic reviews in columns. The following formula was then applied to calculate the CCA. CCA (%) = *N* − *r*/*rc* − *r*: where *N* = number of included publications (sum of checked boxes), *r* = number of rows (primary studies), and *c* = number of columns (number of systematic reviews). CCA less than 5% indicates low overlap and CCA 10% or above indicates moderate/high overlap. The details of the overlap association are presented in Additional file 1: Tables 4–10. Where reviews had a moderate/high overlap (CCA ≥ 10%), decisions regarding which reviews to include were made according to the following criteria:

- Cochrane reviews were given preference over non-Cochrane reviews. Research has highlighted that Cochrane reviews are subject to the least amount of data loss, were of higher quality overall, and were typically more up to date.
- In the case of high overlap between non-Cochrane reviews, preference was assigned as follows:
  1. Highest AMSTAR 2 rating
  2. Most recent
  3. Highest number of studies or participants
- Where a low overlap was observed, both studies were included and compared.

### Update of reviews

The reviews were carefully examined following guidelines by Park et al. to identify whether the reviews needed to be updated (9). A systematic review with meta-analysis was considered out of date if a newly published study led to a change of the result by at least 50%.

### Data synthesis

The included systematic reviews were presented in a tabular form to present their characteristics. The results were synthesised both quantitatively and narratively. The summary estimates from the included meta-analysis were presented as odds ratios/risk ratios/hazard ratios in forest plots (3.3.0), and R Studio (12.1) and STATA (18.0) were used for data synthesis [17–19].

### Patient and public involvement

PPIE representatives (RP and NM) were involved in formulating the research question and study design. They also played key roles in collaboration with clinicians and researchers to agree on the list of pregnancy complications/risk factors and types of cancers to include in the study. NM attended regular study meetings.

## Results

**Fig. 1:**
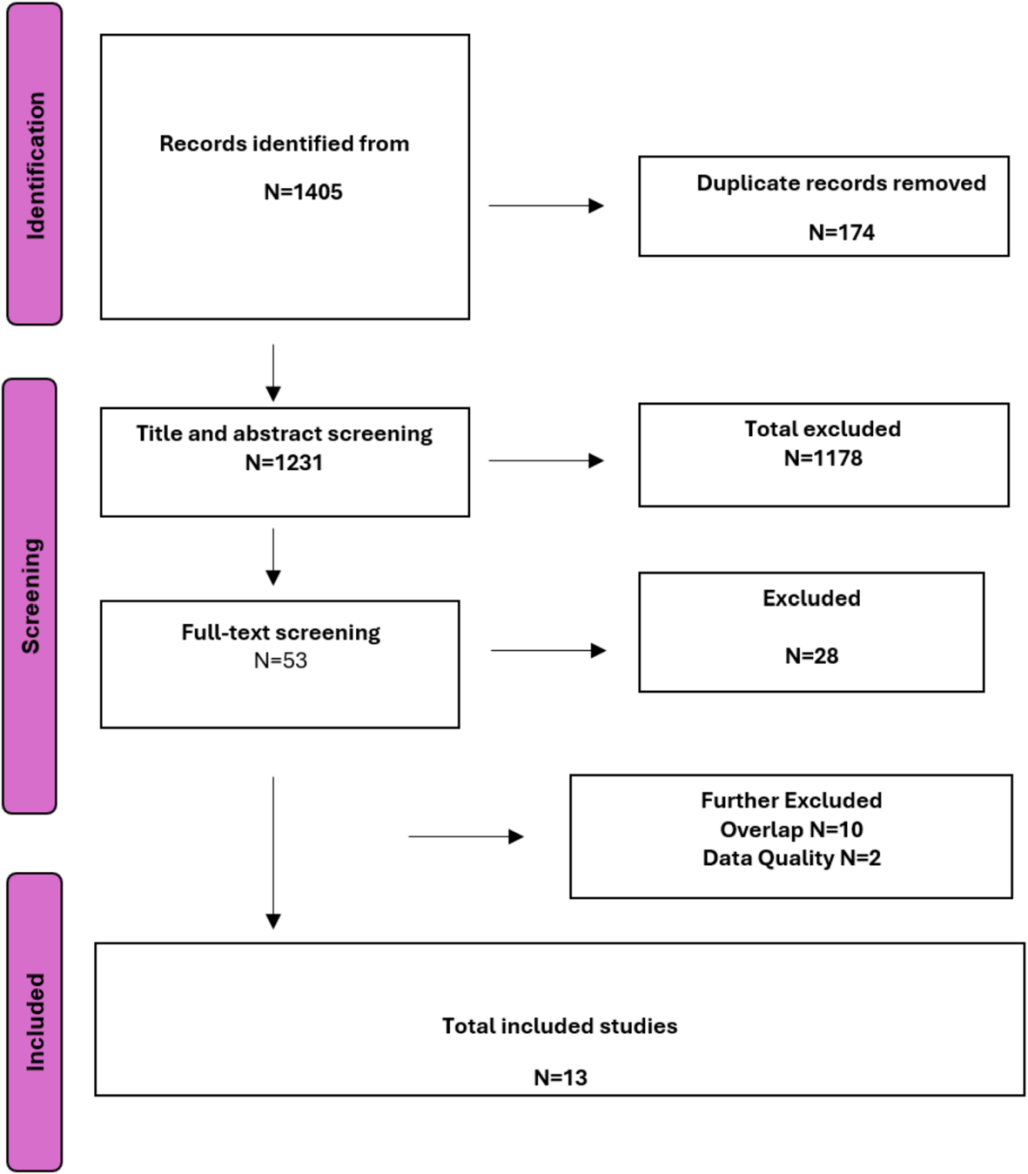
Preferred Reporting Items for Systematic review and Meta-Analysis (PRISMA) flow diagram

The literature search identified 1405 potential reviews. One hundred seventy-four duplicates were excluded. After screening titles and abstracts, 53 reviews were selected for full-text screening, of which 28 were then excluded. Ten were excluded due to overlap and two were excluded due to critically low quality. Thirteen reviews were included consisting of 170 primary studies. Figure 1 shows the selection process in accordance with the Preferred Reporting Items for Systematic review and Meta-Analysis (PRISMA) flow diagram. The characteristics of the included studies are presented in Table 2.

**Table 2:** Characteristics of the included systematic reviews reporting the association of pregnancy complications and risk of cancer

Exposure	Outcomes	Author/year	Number of included studies	Quality
Miscarriage	Ovarian cancer	Dick 2009	2	Low
	Thyroid cancer	Mannathazhathu 2019	10	Moderate
	Breast cancer	Tong 2020	14	Moderate
Miscarriage or induced abortion	Liver cancer	Zhong 2016	4	Moderate
Molar pregnancy	Choriocarcinoma	Albright 2020	19	Moderate
Pre-eclampsia	Any cancer	Bellamy 2007	4	Moderate
	Breast cancer, uterus-related cancer, ovarian cancer	Wang F 2021	27	Moderate
	Uterus-related cancer	Jordao 2023	7	High
	Breast cancer	Yao Min 2023	11	Moderate
GDM	Blood cancer Brain cancer Breast cancer Cervical cancer Colon cancer Endometrial cancer Gastric cancer Lung/bronchial cancer Pancreatic cancer Skin cancer Thyroid cancer Urological cancer Uterine cancer	Wang P 2020	23	Moderate
Delivery mode	Cervical cancer	Douligeris 2022	8	Moderate
Preterm birth	Breast cancer	Razavi 2023	13	Moderate
Multiple birth	Breast cancer	Veisi 2023	10	High

*GDM* gestational diabetes mellitus

### Methodological quality

Quality assessment summary findings are presented in Table 2. Detailed quality assessment of individual reviews is provided in Additional file 1: Table 3. Of 25 reviews assessed for quality (before 10 were excluded due to overlap), two reviews were assessed as high quality [20, 21]. Twelve reviews were assessed as moderate quality [22–33] and nine as low quality [14, 34–41] as they performed poorly across three or more of the critical domains. All the low-quality reviews either failed to include appropriate information on a protocol, study selection, data extraction, and risk of bias. Two reviews were critically low in quality and were excluded [42, 43].

### Overlapping and non-overlapping associations

Of the 25 eligible reviews, overlap in studies was observed in 15 reviews [14, 20–23, 30–32, 35–41], including GDM and breast cancer (*n* = 7), GDM and thyroid cancer (*n* = 2), GDM and pancreatic cancer (*n* = 2), GDM and ovarian cancer (*n* = 2), pre-eclampsia and breast cancer (*n* = 6), pre-eclampsia and endometrial cancer (*n* = 2), and multiple births and breast cancer (*n* = 3). Ten reviews had high overlap and were excluded. The citation matrices detailing the degree of overlap are included in Additional file 1: Tables 3–9.

### Summary findings

The 13 included reviews reported the association of seven pregnancy complications/risk factors with 17 types of cancers. The seven pregnancy complications/risk factors (exposures) were miscarriage, molar pregnancy, pre-eclampsia, GDM, caesarean section, preterm birth, and multiple birth [20, 21, 24–34]. The definition of each exposure (where specified) as used in relevant reviews has been added in Additional file 1: Table 11.

#### Miscarriage

One review including 14 primary studies reported miscarriage to have no association with breast cancer with RR 1.02 (95% CI 0.94–1.12) [29]. This review further reported the association between the number of miscarriages and the risk of breast cancer among nulliparous women, with 10 studies reporting on the relationship between a single miscarriage and risk of breast cancer: pooled RR of 1.08 (0.96–1.22). A further study of the relationship between miscarriage and breast cancer among those with two or more miscarriages reported a pooled RR of 0.94 (0.78–1.14). Dick et al. reported no association between miscarriage and ovarian cancer with OR 0.95 (0.82–1.09) [34]. A review with meta-analysis of seven observational studies reported a significant increased risk of thyroid cancer after miscarriage: OR 1.29 (1.15–1.44) [27].

No significant association between miscarriage or induced abortion and liver cancer risk was found (RR for miscarriage 1.10 (0.71–1.70) 2 studies; RR for induced abortion 1.27 (0.76–2.14) 2 studies) [33].

#### Molar pregnancy

There was more than a four-fold higher risk of developing gestational trophoblastic neoplasia after molar pregnancy in a meta-analysis of 19 studies; OR 4.72 (1.81–12.32). Overall incidence of gestational trophoblastic neoplasia was reported at 15.7% after complete molar pregnancy (1354/8611, 95% CI 15.0–16.5%) and 3.95% after partial molar pregnancy (221/5593, 95% CI 3.47–4.50%) [24].

#### Pre-eclampsia

There was no significant association between pre-eclampsia and uterine cancer; HR 1.07 (0.79–1.46) from one review [20]. Another review including three studies reported a significant association between pre-eclampsia and ovarian cancer (RR 1.82, 1.16–2.85). The association with overall cancer was not significant, RR 0.98 (0.85–1.12) [30]. One review where follow-up of the studies ranged from 8 to 29.2 years reported an inverse association between pre-eclampsia and breast cancer, RR 0.89 (0.83–0.95) [32]. Another study reported no significant association between pre-eclampsia and development of ovarian, uterine, or breast cancer collectively with RR 0.96 (0.73–1.27) [25].

#### Gestational diabetes mellitus (GDM)

One review reported no significant association between GDM and the following cancers: brain, RR 1.26 (0.80–1.97); breast, RR 1.02 (0.87–1.21); colon, RR 1.41 (0.90–2.21); colorectal, RR 1.16 (0.95–1.41); ovarian, RR 1.14 (0.90–1.44); pancreatic, RR 3.49 (0.80–15.23); skin, RR 1.13 (0.81–1.56); uterine, RR 1.02 (0.81–1.29); urological, RR 0.98 (0.73–1.31). A significant association was reported with blood cancer, RR 1.48 (1.04–2.09); liver cancer, RR 1.27 (1.03–1.55); stomach cancer, RR 1.43 (1.02–2.00); and thyroid cancer, RR 1.28 (1.16–1.42) [31].

#### Caesarean section

A meta-analysis of eight observational studies reported no association between a previous caesarean section and development of cervical cancer, RR 1.33 (0.83–2.15) [26].

#### Preterm birth

One review reported a significant association between early gestation at birth and breast cancer with OR 1.03 (1.00–1.07). The risk was significantly increased in women who delivered at 37–39 weeks of gestation (RR 1.03, 1.01–1.06) and 26–31 weeks of gestation (RR 1.25, 1.04–1.47) compared to women who delivered at 40–41 weeks of gestation [28].

#### Multiple birth

**Fig. 2:**
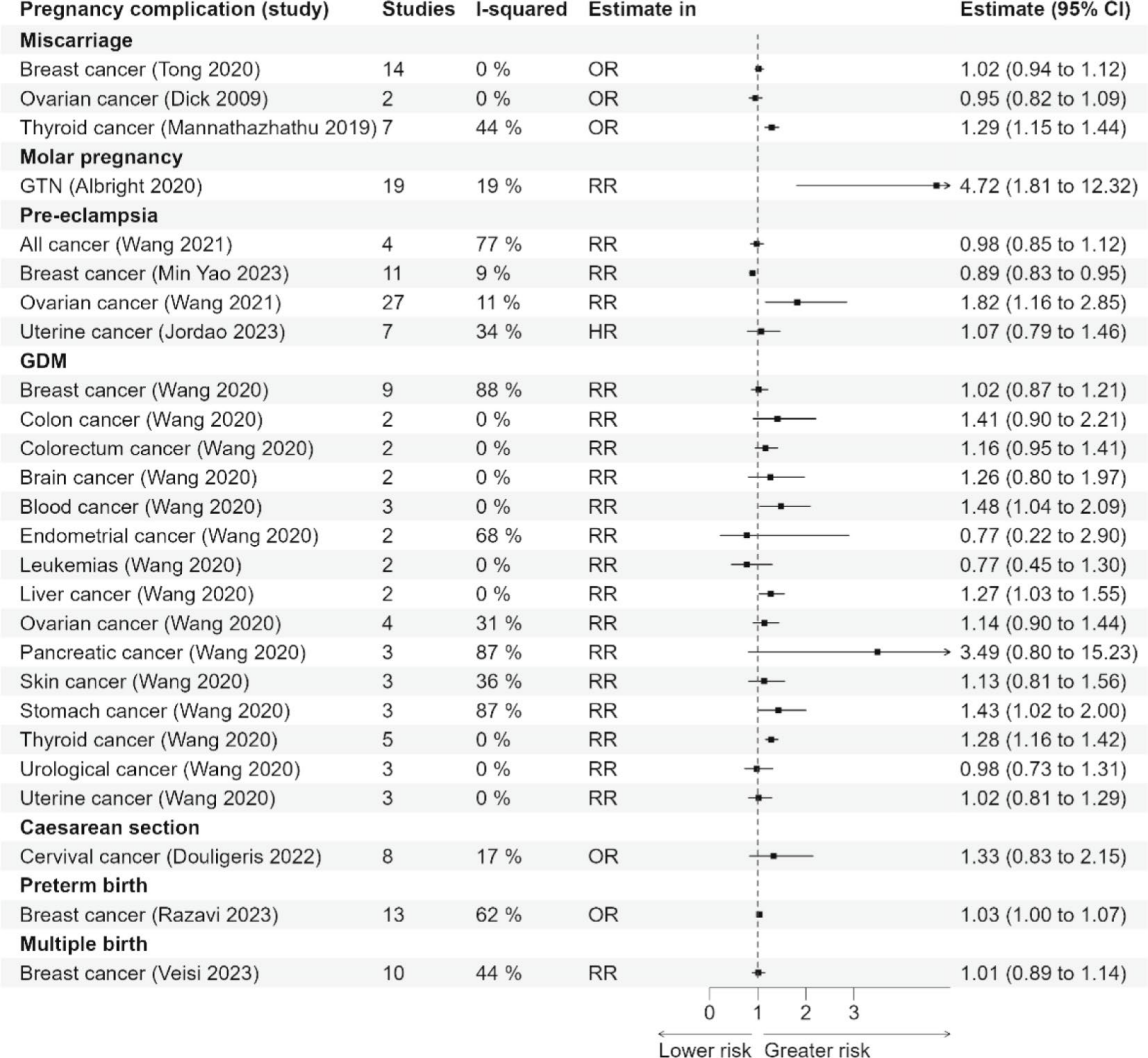
Forest plot association of pregnancy complications/risk factors and development of cancers*.* GTN gestational trophoblastic neoplasia, GDM gestational diabetes mellitus, OR odds ratio, RR risk ratio, HR hazards ratio

In a meta-analysis of 19 studies (11 case–control studies and 8 cohort studies), there was no significant association between multiple pregnancies and breast cancer, RR 1.01 (0.89–1.14) in cohort studies and OR 0.89 (0.83–0.95; I2 41.73%, *P* 0.07) in case–control studies, respectively (Fig. 2) [21].

## Discussion

### Summary of results by cancer types

Overall, the evidence did not indicate a consistent increase in breast cancer risk following pregnancy complications. Most exposures, including miscarriage and gestational diabetes mellitus, showed no clear association with breast cancer, while pre-eclampsia was associated with a modest reduction in risk. Risk of ovarian cancer was higher following pre-eclampsia. Positive associations between GDM and haematological, liver, stomach, and thyroid cancers were identified.

Cases of gestational trophoblastic neoplasia are rare but are known to occur following molar pregnancy, as identified in this review. This condition was four and a half times more likely after complete molar compared to partial molar pregnancies and most of the identified cases occurred outside of the current recommended follow-up period following human chorionic gonadotropin (hCG) normalisation.

No systematic reviews have been reported which explore an association between stillbirth and later cancer, although some primary studies have noted an 18% increased risk [44]. No reviews reported on cancer risk following placental disorders, hyperemesis gravidarum, obstetric haemorrhage, post-partum depression, puerperal psychosis, perineal trauma, obstetric cholestasis, or pelvic girdle pain.

### Strengths, limitations, and methodological issues

This umbrella review was designed to operationalise a published protocol which was standardised across five outcome (autoimmune diseases, functional disorders, metabolic disorder, cancer, mental health conditions) domains [13]. A rigorous framework in following Joanna Briggs Institute (JBI) umbrella review methodology was applied [1]. The Preferred Reporting Items for Overviews of Reviews (PRIOR) checklist was used to report the review. The design and application of the search strategy was the result of a comprehensive expert consensus review and iterative development of search terms.

Several limitations should be considered when interpreting these findings.

During data extraction, incomplete reporting in some systematic reviews limited full reporting of key study characteristics such as the number of participants, follow-up duration, and definitions of exposures or outcomes. Defining appropriate comparator groups was also challenging, as many studies did not clearly specify whether controls comprised women without the pregnancy complication of interest, or this was only implied from the study context. These limitations may affect the interpretation of the findings. Furthermore, as this umbrella review synthesised evidence from published systematic reviews and meta-analyses, it was not possible to standardise exposure definitions, outcome ascertainment, or confounder adjustment across primary studies. Assessment of potential sources of bias therefore relied on the reporting and risk-of-bias evaluations conducted within the included reviews, a limitation widely acknowledged in umbrella reviews and reflective of broader inconsistencies in reporting quality within observational research.

### Interpretation of findings

These findings suggest that pregnancy complications are unlikely to represent strong predictors of later breast cancer risk, although the inverse association observed with pre-eclampsia warrants further investigation into possible biological mechanisms.

The review findings suggest that pregnancy may act as a ‘stress test’ for later health, identifying women at increased risk of future cancer, particularly those who have experienced molar pregnancy or GDM. It is less clear whether complications of pregnancy play a causative role in future disease.

Potential underlying mechanisms for the associations identified between GDM and various cancers may be linked to insulin resistance and/or hyperinsulinaemia, or *Helicobacter pylori* infection as all have been implicated in malignant cell growth [31, 45]. As the prevalence of *H. pylori* infection varies geographically and is higher in certain regions, including parts of Asia where stomach cancer incidence is also elevated, regional differences in infection rates may partly influence the observed associations between GDM and stomach cancer. However, the included reviews did not consistently report geographic variation, and therefore this possibility could not be formally evaluated in the current analysis. GDM is also more common among individuals with increased BMI, a group at higher risk of subsequent metabolic dysfunction, chronic liver disease including cirrhosis, and cancer, which may partly explain the observed associations. Inflammation levels may also play a role, as these are recognised to be key mediators in the development of GDM [17, 46]. The association between molar pregnancy and gestational trophoblastic neoplasia is well established and reflects progression within the trophoblastic disease spectrum rather than the development of an unrelated long-term malignancy. Other shared mechanisms may exist between pregnancy complications and cancer development. For example, endometriosis has been associated with both pre-eclampsia and increased ovarian cancer risk, although it is unlikely to fully explain the specific cancer subtypes observed in this review.

### Implications for practice and public health

Positive associations between pregnancy complications and cancer risk were limited to specific cancer types, including liver, ovarian, thyroid, stomach, and haematological cancers, as well as gestational trophoblastic neoplasia. While these findings may contribute to a broader understanding of long-term maternal health risks, the evidence remains heterogeneous and should be interpreted cautiously.

Strengthening communication between maternity services and primary care regarding pregnancy complications may support broader long-term health monitoring for women. Such approaches are already recommended for women with a history of pre-eclampsia or gestational diabetes due to their increased risk of future hypertension and cardiovascular disease, prompting early lifestyle advice and follow-up screening in primary care [47].

### Implications for future research

The findings of this review have highlighted significant gaps and limitations of data exploring pregnancy complications and risk of cancer, including inconsistent reporting of studies. The findings suggesting that risk of liver, thyroid, stomach, and haematological cancers are increased for women after GDM warrant further investigation to better understand causal mechanisms. Future research could explore how long-term management of women who develop GDM or pre-eclampsia affects the relationship with the relevant cancer outcomes.

This area of investigation is currently in its infancy. Studies exploring the role of additional pregnancy complications and cancer outcomes are warranted. These could include obstetric cholestasis, placental disorders, hyperemesis gravidarum, or obstetric haemorrhage.

Given the associations between pre-eclampsia and ovarian cancer as well as GDM and liver cancer, it may be appropriate to explore the role of these pregnancy complications in prediction models for the respective cancers. Additionally, although not included in the current review, risk prediction for clear cell carcinoma arising from abdominal wall endometriosis following caesarean section may also be beneficial due to the poor overall survival following delayed diagnosis and high degree of invasiveness.

A substantial number of potential confounders were identified in the included studies, including missing or unreported variables such as age, parity, lifestyle factors (e.g. smoking, alcohol consumption, BMI), age at birth, previous births, and prior pregnancy complications. Furthermore, it was challenging to identify studies that used unexposed women as a comparator. National datasets and registries could help address these gaps and allow further testing of these associations.

Large studies with significant follow-up periods would refine and strengthen existing knowledge as well as provide robust methodological design for research into current gaps in evidence. It is possible that findings are not global and western lifestyle factors may be salient variables. Considering this in future research would assist in informing lifestyle education programmes to reduce potential risk of disease.

## Conclusions

GDM was positively associated with thyroid, stomach, liver, and haematological cancers, while pre-eclampsia was positively associated with ovarian cancer but inversely associated with breast cancer. This umbrella review highlights both positive and negative associations between pregnancy complications and subsequent cancer risk. Future research should investigate additional pregnancy complications and risk factors and clarify the underlying mechanisms linking GDM and pre-eclampsia with cancer in later life.

## Supplementary Information

Additional file 1: Table 1. PRIOR checklist. Table 2. Search strategy in MEDLINE. Table 3. Quality assessment of the reviews NOS scale. Table 4. Corrected covered area Matrix GDM and breast cancer. Table 5. Corrected covered area Matrix GDM and thyroid cancer. Table 6. Corrected covered area Matrix GDM and pancreatic cancer. Table 7. Corrected covered area Matrix Pre-eclampsia and breast cancer. Table 8. Corrected covered area Matrix Pre-eclampsia and endometrial cancer. Table 9. Corrected covered area Matrix Twin births and breast cancer. Table 10. Corrected covered area summary. Table 11. Exposure definitions as used in the reviews.

## Data Availability

Not applicable.
