# Supplementary material for: The association of pregnancy complications/risk factors with the development of cancer in women: an umbrella review
Source: BMC Med. 2026 Apr 13;24:313. doi: 10.1186/s12916-026-04844-6 (PMC13185374; doi:10.1186/s12916-026-04844-6)
Supplement: Supplementary file 1 — Additional file 1: Table 1. PRIOR checklist. Table 2. Search strategy in MEDLINE. Table 3. Quality assessment of the reviews NOS scale. Table 4. Corrected covered area Matrix GDM and breast cancer. Table 5. Corrected covered area Matrix GDM and thyroid cancer. Table 6. Corrected covered area Matrix GDM and pancreatic cancer. Table 7. Corrected covered area Matrix Pre-eclampsia and breast cancer. Table 8. Corrected covered area Matrix Pre-eclampsia and endometrial cancer. Table 9. Corrected covered area Matrix Twin births and breast cancer. Table 10. Corrected covered area summary. Table 11. Exposure definitions as used in the reviews. [file 12916_2026_4844_MOESM1_ESM.docx]

Contents

[*Table 1 PRIOR checklist(Preferred Reporting Items for Overviews of Reviews* 1](#_Toc225745498)

[*Table 2 Search strategy in MEDLINE* 6](#_Toc225745499)

[Table 3 Quality assessment of the reviews AMSTAR 2 scale 10](#_Toc225745500)

[Table 4 Corrected covered area Matrix- GDM and breast cancer 11](#_Toc225745501)

[Table 5 Corrected covered area Matrix GDM and thyroid cancer 12](#_Toc225745502)

[Table 6 Corrected covered area Matrix GDM and pancreatic cancer 12](#_Toc225745503)

[Table 7 Corrected covered area Matrix Pre-eclampsia and breast cancer 13](#_Toc225745504)

[Table 8 Corrected covered area Matrix Pre-eclampsia and endometrial cancer 14](#_Toc225745505)

[Table 9 Corrected covered area Matrix Twin births and breast cancer 14](#_Toc225745506)

[Table 10 Corrected covered area summary 15](#_Toc225745507)

[Table 11 Exposure definitions as used in the reviews 16](#_Toc225745508)

# *Table 1 PRIOR checklist(Preferred Reporting Items for Overviews of Reviews*

| Section |  | |  |  |
| --- | --- | --- | --- | --- |
| topic | Item No | | Item | Location where item is reported |
| **Title** |  | |  |  |
| Title | 1 | | Identify the report as an overview of reviews. | Line 1 |
| **Abstract** |  | |  |  |
| Abstract | 2 | | Provide a comprehensive and accurate summary of the purpose, methods, and results of the overview of reviews. | Line 16 to 45 |
| **Introduction** |  | |  |  |
| Rationale | 3 | | Describe the rationale for conducting the overview of reviews in the context of existing knowledge. | Line 46  background |
| Objectives | 4 | | Provide an explicit statement of the objective(s) or question(s) addressed by the overview of reviews. | Line 83-93 |
| Methods |  | |  |  |
| Eligibility criteria | 5a | | Specify the inclusion and exclusion criteria for the overview of reviews. If supplemental primary studies were included, this should be stated, with a rationale. | Inclusion and exclusion criteria  Line 103-133 |
|  | 5b | | Specify the definition of “systematic review” as used in the inclusion criteria for the overview of reviews. | Line 124-126 |
| Information sources | 6 | | Specify all databases, registers, websites, organisations, reference lists, and other sources searched or consulted to identify systematic reviews and supplemental primary studies (if included). Specify the date when each source was last searched or consulted. | Search strategy  Line 134-141 |
| Search strategy | 7 | | Present the full search strategies for all databases, registers and websites, such that they could be reproduced. Describe any search filters and limits applied. | Search strategy  Line 134-141  Additional file table 3 |
| Selection process | 8a | | Describe the methods used to decide whether a systematic review or supplemental primary study (if included) met the inclusion criteria of the overview of reviews. | Study selection  Line 142-153 |
|  | 8b | | Describe how overlap in the populations, interventions, comparators, and/or outcomes of systematic reviews was identified and managed during study selection. | Line 178-186 and  Additional file text 1 |
| Data collection process | 9a | | Describe the methods used to collect data from reports. | Data extraction  Line 154-164 |
|  | 9b | | If applicable, describe the methods used to identify and manage primary study overlap at the level of the comparison and outcome during data collection. For each outcome, specify the method used to illustrate and/or quantify the degree of primary study overlap across systematic reviews. | Line 178-186  Additional file text 1, table 7 and 8 |
|  | 9c | | If applicable, specify the methods used to manage discrepant data across systematic reviews during data collection. | Data extraction  Line 154-164 |
| Data items | 10 | | List and define all variables and outcomes for which data were sought. Describe any assumptions made and/or measures taken to identify and clarify missing or unclear information. | Data extraction  Line 154-164  And Additional file table 5 |
| Risk of bias assessment | 11a | | Describe the methods used to assess risk of bias or methodological quality of the included systematic reviews. | Quality assessment  Line 167 |
|  | 11b | | Describe the methods used to collect data on (from the systematic reviews) and/or assess the risk of bias of the primary studies included in the systematic reviews. Provide a justification for instances where flawed, incomplete, or missing assessments are identified but not reassessed. | Quality assessment  Line 165-177 |
|  | 11c | | Describe the methods used to assess the risk of bias of supplemental primary studies (if included). | NA |
| Synthesis methods | 12a | | Describe the methods used to summarise or synthesise results and provide a rationale for the choice(s). | Data synthesis  Line 206-224 |
|  | 12b | | Describe any methods used to explore possible causes of heterogeneity among results. | Data synthesis  Line 206-224 |
|  | 12c | | Describe any sensitivity analyses conducted to assess the robustness of the synthesised results. | NA |
| Reporting bias assessment | 13 | | Describe the methods used to collect data on (from the systematic reviews) and/or assess the risk of bias due to missing results in a summary or synthesis (arising from reporting biases at the levels of the systematic reviews, primary studies, and supplemental primary studies, if included). | Data synthesis  Line 187-202 |
| Certainty assessment | 14 | | Describe the methods used to collect data on (from the systematic reviews) and/or assess certainty (or confidence) in the body of evidence for an outcome. | Data synthesis  Line 206-224 |
| Results |  |  | |  |
| Systematic review and supplemental primary study selection | 15a | Describe the results of the search and selection process, including the number of records screened, assessed for eligibility, and included in the overview of reviews, ideally with a flow diagram. | | Results  Line 209 onwards and  PRISMA flowchart |
|  | 15b | Provide a list of studies that might appear to meet the inclusion criteria, but were excluded, with the main reason for exclusion. | | Additional file table 4 |
| Characteristics of systematic reviews and supplemental primary studies | 16 | Cite each included systematic review and supplemental primary study (if included) and present its characteristics. | | Table 2 main document and Additional file table 11,12 ,13 |
| Primary study overlap | 17 | Describe the extent of primary study overlap across the included systematic reviews. | | Line 178-186  Additional file text 1, table 7 and 8 |
| Risk of bias in systematic reviews, primary studies, and supplemental primary studies | 18a | Present assessments of risk of bias or methodological quality for each included systematic review. | | Quality assessment  Line 165-177 Additional file table 6 |
|  | 18b | Present assessments (collected from systematic reviews or assessed anew) of the risk of bias of the primary studies included in the systematic reviews. | | Additional file table 11 |
|  | 18c | Present assessments of the risk of bias of supplemental primary studies (if included). | | NA |
| Summary or synthesis of results | 19a | For all outcomes, summarise the evidence from the systematic reviews and supplemental primary studies (if included). If meta-analyses were done, present for each the summary estimate and its precision and measures of statistical heterogeneity. If comparing groups, describe the direction of the effect. | | Summary of the results  Line 282 onwards  Figures 2 and 3 and table 3 |
|  | 19b | If meta-analyses were done, present results of all investigations of possible causes of heterogeneity. | | Additional file text 1 |
|  | 19c | If meta-analyses were done, present results of all sensitivity analyses conducted to assess the robustness of synthesised results. | | NA |
| Reporting biases | 20 | Present assessments (collected from systematic reviews and/or assessed anew) of the risk of bias due to missing primary studies, analyses, or results in a summary or synthesis (arising from reporting biases at the levels of the systematic reviews, primary studies, and supplemental primary studies, if included) for each summary or synthesis assessed. | | Line 466-474  Additional file text 1 |
| Certainty of evidence | 21 | Present assessments (collected or assessed anew) of certainty (or confidence) in the body of evidence for each outcome. | | Strengths and limitations  Line 449 |
| **Discussion** |  |  | |  |
| Discussion | 22a | Summarise the main findings, including any discrepancies in findings across the included systematic reviews and supplemental primary studies (if included). | | Line 448-458  Table 3 |
|  | 22b | Provide a general interpretation of the results in the context of other evidence. | | Conclusion  Line 514-417 |
|  | 22c | Discuss any limitations of the evidence from systematic reviews, their primary studies, and supplemental primary studies (if included) included in the overview of reviews. Discuss any limitations of the overview of reviews methods used. | | Strength and limitation  Line 449 |
|  | 22d | Discuss implications for practice, policy, and future research (both systematic reviews and primary research). Consider the relevance of the findings to the end users of the overview of reviews, eg, healthcare providers, policymakers, patients, among others. | | Line 497-513 |
| **Other information** |  |  | |  |
| Registration and protocol | 23a | Provide registration information for the overview of reviews, including register name and registration number, or state that the overview of reviews was not registered. | | Methods line 101 |
|  | 23b | Indicate where the overview of reviews protocol can be accessed, or state that a protocol was not prepared. | | Methods line 101 |
|  | 23c | Describe and explain any amendments to information provided at registration or in the protocol. Indicate the stage of the overview of reviews at which amendments were made. | | Additional file table 2 |
| Support | 24 | Describe sources of financial or non-financial support for the overview of reviews, and the role of the funders or sponsors in the overview of reviews. | | Line 525 |
| Competing interests | 25 | Declare any competing interests of the overview of reviews' authors. | | Line 530 |
| Author information | 26a | Provide contact information for the corresponding author. | | Line 12 all |
|  | 26b | Describe the contributions of individual authors and identify the guarantor of the overview of reviews. | | Line 519-523 |
| Availability of data and other materials | 27 | Report which of the following are available, where they can be found, and under which conditions they may be accessed: template data collection forms; data collected from included systematic reviews and supplemental primary studies; analytic code; any other materials used in the overview of reviews. | | Additional file |

# *Table 2 Search strategy in MEDLINE*

| Ovid MEDLINE(R) ALL <1946 to May 03, 2024> |  |
| --- | --- |
|  |  |
| 1 | pregnancy complication*.mp. or exp Pregnancy Complications/ |
| 2 | exp Abortion, Spontaneous/ or ((recurrent adj3 miscarr$) or miscarr$ or early pregnancy loss$).mp. |
| 3 | (stillbirth or still birth).mp. or exp Stillbirth/ or exp Fetal Death/ or (f?etal death$ or f?etal demise$).mp. |
| 4 | Hypertension, Pregnancy-Induced/ or (gestational hypertension or (pregnancy adj3 hypertensi$)).mp. |
| 5 | (preeclampsia or pre-eclampsia).mp. or exp Pre-Eclampsia/ |
| 6 | exp Eclampsia/ or (eclampsia or tox?emia).mp. |
| 7 | HELLP.mp. or exp HELLP Syndrome/ |
| 8 | placenta accreta.mp. or exp Placenta Accreta/ or placenta percreta.mp. or placenta increta.mp. or morbidly adherent placenta.mp. or abnormally invasive placenta.mp. |
| 9 | Placenta$ abruption.mp. or exp Abruptio Placentae/ |
| 10 | Placenta pr?evia.mp. or exp placenta previa/ or low lying placenta.mp. |
| 11 | Hyperemesis Gravidarum.mp. or Hyperemesis Gravidarum/ or morning sickness.mp. or exp Morning Sickness/ |
| 12 | (((pregnan$ or gestation$ or prenatal$ or antenatal$ or pre-natal$ or ante-natal$ or maternal$) adj2 diabet$) or gestational diabetes).mp. or exp Diabetes, Gestational/ or GDM.mp. |
| 13 | ectopic pregnancy.mp. or exp Pregnancy, Ectopic/ or ((tub$$ adj3 pregnanc$) or (cornual adj3 pregnanc$) or (heterotopic adj3 pregnanc$) or (abdomin$ adj3 pregnanc$) or (extrauterine adj3 pregnanc$) or (interstitial adj3 pregnanc$) or (cervi$ adj3 pregnanc$) or (ovar$ adj3 pregnanc$) or (cesarean scar adj3 pregnanc$)).mp. |
| 14 | exp Gestational Trophoblastic Disease/ or gestational trophoblastic.mp. or exp Hydatidiform Mole/ or ((hydatid? adj2 mole?) or (molar adj2 pregnanc?)).mp. |
| 15 | exp Choriocarcinoma/ or choriocarcinoma.mp. |
| 16 | exp Pregnancy, Multiple/ or ((pregnanc* or gestation*) adj (twin* or triplet* or quadruplet* or quintuplet* or multiple or multi?f?et*)).mp. or (Monochorionic or dichorionic).mp. |
| 17 | exp Postpartum Hemorrhage/ or (postpartum hemorrhage or post partum hemorrhage or postpartum haemorrhage or post partum haemorrhage).ti,ab. or obstetric haemorrhage.mp. |
| 18 | obstetric labor, premature.mp. or exp Obstetric Labor, Premature/ or (premature labor or premature labour or preterm labor or preterm labour or preterm birth).mp. |
| 19 | exp Cesarean Section, Repeat/ or cesarean.mp. or exp Cesarean Section/ or (caesarean or cesarean or caesarian or cesarian or cesarien or caesarien or c-section or c section).mp. |
| 20 | exp Extraction, Obstetrical/ or exp Obstetrical Forceps/ or ((operative or instrumental or assisted or forcep* or ventouse* or vacuum*) adj1 (deliver* or birth*)).mp. |
| 21 | low birth weight.mp. or exp Infant, Low Birth Weight/ or (low birth weight* adj4 very low birth weight*).mp. |
| 22 | exp Infant, Small for Gestational Age/ or small for gestational age.mp. or (small adj3 gestational age).mp. |
| 23 | (intra?uterine growth adj2 (restriction* or retardation)).mp. or iugr.ti,ab. |
| 24 | fetal growth retardation.mp. or exp Fetal Growth Retardation/ or (fetal growth adj2 (restriction? or retardation)).mp. |
| 25 | postpartum depression.mp. or exp Depression, Postpartum/ |
| 26 | ((postpartum* or post partum* or post-partum* or postnatal* or post natal* or post-natal* or perinatal* or peri natal* or peri-natal* or puerp*) and (depress* or dysthymi* or adjustment disorder* or mood disorder* or affective disorder*)).mp. |
| 27 | (((postpartum* or post partum* or post-partum* or postnatal* or post natal* or post-natal or perinatal* or peri natal* or peri-natal* or puerp*) and (psychos#s or psychotic)) or psychosis after childbirth).mp. |
| 28 | (((third or fourth or 3rd or 4th) adj degree) and tear*).mp. |
| 29 | (((anal near adj2 sphincter) or (rectal adj mucosa) or rectum or (anal adj epithelium) or anus or (recto?vaginal adj2 fistulae) or (anorectal adj mucosa) or (anal adj skin)) and (tear* or injur* or damage* or lacerat* or rupture* or trauma)).mp. [mp=title, book title, abstract, original title, name of substance word, subject heading word, floating sub-heading word, keyword heading word, organism supplementary concept word, protocol supplementary concept word, rare disease supplementary concept word, unique identifier, synonyms, population supplementary concept word, anatomy supplementary concept word] |
| 30 | ((obstetric* and anal and sphincter and injur*) or (anal and sphincter and injur*)).mp. |
| 31 | (exp Pregnancy/ or exp Obstetrics/ or (pregnan* or obstetric*).mp.) and (exp Cholestasis/ or exp Cholestasis, Intrahepatic/) |
| 32 | pelvic girdle pain.mp. or exp Pelvic Girdle Pain/ |
| 33 | (symphysis pubis adj3 (pain$ or dysfunction$)).mp. |
| 34 | Pubic Symphysis Diastasis.mp. or exp Pubic Symphysis Diastasis/ |
| 35 | (Sacroiliac joint dysfunction or PGP).mp. |
| 36 | 1 or 2 or 3 or 4 or 5 or 6 or 7 or 8 or 9 or 10 or 11 or 12 or 13 or 14 or 15 or 16 or 17 or 18 or 19 or 20 or 21 or 22 or 23 or 24 or 25 or 26 or 27 or 28 or 29 or 30 or 31 or 32 or 33 or 34 or 35 |
| 37 | exp Lung Neoplasms/ |
| 38 | lung cancer.mp. |
| 39 | exp Breast Neoplasms/ |
| 40 | breast cancer.mp. |
| 41 | exp Uterine Cervical Neoplasms/ |
| 42 | cancer cervix.mp. |
| 43 | cervical cancer.mp. |
| 44 | cervical neoplasms.mp. |
| 45 | cer$ cancer.mp. |
| 46 | Neoplasms, Unknown Primary/ |
| 47 | cancer of unknown primary.mp. |
| 48 | exp Pancreatic Neoplasms/ |
| 49 | cancer pancrea$.mp. |
| 50 | Carcinoma, Pancreatic Ductal/ |
| 51 | pancreas$ carcinoma.mp. |
| 52 | exp Ovarian Neoplasms/ |
| 53 | ((ovarian or ovary or ovar$) adj2 carcinoma).tw. |
| 54 | exp Uterine Neoplasms/ |
| 55 | Endometrial neoplasms/ |
| 56 | cancer, uter$.mp. |
| 57 | ((uterine or uterus or uter$) adj2 carcinoma).tw. |
| 58 | exp Esophageal Neoplasms/ |
| 59 | ((esoph$ or oesoph$) adj3 (carcinoma or cancer)).tw. |
| 60 | exp Central Nervous System Neoplasms/ |
| 61 | brain cancer.mp. |
| 62 | exp Liver Neoplasms/ |
| 63 | liver cancer.mp. |
| 64 | exp Skin neoplasms/ |
| 65 | melanoma skin cancer.mp. |
| 66 | melanoma.mp. |
| 67 | exp Kidney Neoplasms/ |
| 68 | kidney cancer.mp. |
| 69 | Carcinoma, Renal Cell/ |
| 70 | renal cancer.mp. |
| 71 | ((kidney or rena? or neph$) adj3 (carcinoma or cancer or neoplas?)).tw. |
| 72 | exp Thyroid Neoplasms/ |
| 73 | Thyroid cancer.mp. |
| 74 | thyroid carcinoma.mp. |
| 75 | Leukemia/ |
| 76 | leukemia.mp. |
| 77 | leukaemia.mp. |
| 78 | exp Colorectal Neoplasms/ |
| 79 | colorectal cancer.mp. |
| 80 | colon cancer.mp. or exp Colonic Neoplasms/ |
| 81 | exp Lymphoma/ |
| 82 | lymphoma.mp. |
| 83 | 37 or 38 or 39 or 40 or 41 or 42 or 43 or 44 or 45 or 46 or 47 or 48 or 49 or 50 or 51 or 52 or 53 or 54 or 55 or 56 or 57 or 58 or 59 or 60 or 61 or 62 or 63 or 64 or 65 or 66 or 67 or 68 or 69 or 70 or 71 or 72 or 73 or 74 or 75 or 76 or 77 or 78 or 79 or 80 or 81 or 82 |
| 84 | 36 and 83 |
| 85 | (systematic$ adj2 (review$ or overview)).ti,ab. |
| 86 | (systematic$ adj5 review$).tw,sh. |
| 87 | meta-analysis.mp. or exp meta-analysis/ |
| 88 | 85 or 86 or 87 |
| 89 | 84 and 88 |

# Table 3 Quality assessment of the reviews AMSTAR 2 scale

| Reviews | 1 | 2 | 3 | 4 | 5 | 6 | 7 | 8 | 9 | 10 | 11 | 12 | 13 | 14 | 15 | 16 |  |
| --- | --- | --- | --- | --- | --- | --- | --- | --- | --- | --- | --- | --- | --- | --- | --- | --- | --- |
| Albrigh 2020 | Yes | Partial Yes | Yes | Yes | Yes | Yes | Yes | No | Yes | No | Yes | No | No | No | Yes | No | Moderate |
| Bellamy 2007 | Yes | Partial Yes | Yes | Partial yesYes | Yes | Yes | Yes | No | Yes | No | Yes | Yes | Yes | Yes | Yes | Yes | Moderate |
| Dick 2009 | Yes | Partial Yes | Yes | Yes | Yes | Yes | Yes | No | Yes | No | Yes | Yes | No | No | Yes | No | Low |
| Douligeris 2022 | Yes | Partial Yes | Yes | Yes | Yes | Yes | Yes | Yes | Yes | No | Yes | No | Yes | Yes | Yes | Yes | Moderate |
| Jordao 2023 | Yes | Yes | Yes | Yes | Yes | Yes | No | Yes | Yes | Yes | Yes | Yes | Yes | Yes | Yes | Yes | High |
| Mannathazhathu 2019 | Yes | Yes | No | Yes | Yes | Yes | Yes | Yes | Yes | No | Yes | Yes | Yes | Yes | Yes | Yes | Moderate |
| Razavi 2023 | Yes | Yes | No | Yes | Yes | Yes | Yes | Yes | Yes | No | Yes | Yes | Yes | Yes | Yes | Yes | Moderate |
| Tong 2020 | Yes | Yes | Yes | Partial Yes | Yes | Yes | No | Yes | Yes | Yes | Yes | Yes | No | Yes | Yes | Yes | Moderate |
| Veisi 2023 | Yes | Yes | Yes | Yes | Yes | Yes | Yes | Yes | Yes | Yes | Yes | Yes | Yes | Yes | Yes | Yes | High |
| Wang F 2021 | Yes | Yes | Yes | Yes | Yes | Yes | Yes | Yes | Yes | Yes | Yes | Yes | Yes | Yes | Yes | Yes | Moderate |
| Wang P 2020 | Yes | Yes | Yes | Yes | Yes | Yes | Yes | Yes | Yes | Yes | Yes | Yes | Yes | Yes | Yes | Yes | Moderate |
| Yao Min 2023 | Yes | Yes | Yes | Yes | Yes | Yes | Yes | Yes | Yes | Yes | Yes | Yes | Yes | Yes | Yes | Yes | Moderate |
| Zhong 2016 | Yes | Yes | Yes | Yes | Yes | Yes | Yes | Yes | Yes | Yes | Yes | Yes | Yes | Yes | Yes | Yes | Moderate |
| **Overlapping Excluded Reviews** | | | | | | | | | | | | | | | | | |
| Dong 2022 | Yes | Yes | Yes | Yes | Yes | No | No | Partial Yes | Partial Yes | Yes | No  MA | No  MA | No | No | No  MA | Yes | Low |
| Flachs 2022 | Yes | Yes | Yes | Yes | Yes | No | No | Partial Yes | Partial Yes | Yes | No  MA | No  MA | No | No | No  MA | Yes | Low |
| Hardefeldt 2012 | Yes | No | Yes | Yes | Yes | Yes | No | Yes | Yes | Yes | Yes | Yes | Yes | Yes | Yes | Yes | Low |
| Kim H 2012 | Yes | No | Yes | Partial Yes | Yes | Yes | No | Yes | Yes | Yes | Yes | Yes | Yes | Yes | Yes | Yes | Low |
| Kim J 2013 | Yes | No | Yes | Partial Yes | Yes | Yes | No | Yes | Yes | No | Yes | Yes | No | Yes | Yes | Yes | Low |
| Sun 2018 | Yes | Yes | Yes | Yes | Yes | Yes | Yes | Yes | Yes | Yes | Yes | Yes | Yes | Yes | Yes | Yes | Moderate |
| Tong 2014 | Yes | Yes | Yes | Yes | Yes | No | No | Partial Yes | Partial Yes | Yes | No  MA | No  MA | No | No | No  MA | Yes | Low |
| Xie 2019 | Yes | Yes | Yes | Yes | Yes | Yes | Yes | Yes | Yes | Yes | Yes | Yes | Yes | Yes | Yes | Yes | Moderate |
| Zohre 2018 | yes | No | yes | Partial Yes | yes | yes | No | partial Yes | partial Yes | No | No meta-analysis conducted | No meta-analysis conducted | Yes | yes | No meta-analysis conducted | No | Low |
| Simon 2021 | yes | No | yes | Partial Yes | yes | yes | No | partial Yes | partial Yes | No | No meta-analysis conducted | No meta-analysis conducted | Yes | yes | No meta-analysis conducted | No | Low |
| **Excluded critically low** | | | | | | | | | | | | | | | | | |
| Slouha 2023 | yes | No | yes | Partial Yes | yes | yes | No | partial Yes | partial Yes | No | No MA | No MA | No | No | No MA | No | Critically Low |
| Brind 1996 | Yes | Partial Yes | Yes | Yes | Yes | Yes | Yes | No | No | No | No MA | No MA | No | No | No MA | No | Critically Low |

# Table 4 Corrected covered area Matrix- GDM and breast cancer

| Primary studies | Flachs 2022  Narrative | Hardefeldt, 2012 | Simon 2021  Narrative | Xie 2019  Meta-analysis | Tong  2014  MA | Wang  2020  MA | Zohreh  2018 |
| --- | --- | --- | --- | --- | --- | --- | --- |
| 1. Ardalan 2012 |  |  | X | X |  | X |  |
| 1. Bejaimal 2016 |  |  | X | X |  | X | X |
| 1. Bertrand 2020 | X |  | X |  |  |  |  |
| 1. Brasky 2013 | X |  | X | X | X | X | X |
| 1. Cnattiongius 2OO5 |  |  |  |  | X |  | X |
| 1. Dawson 2004 |  |  |  |  |  |  | X |
| 1. Fuchs 2017 | X |  | X | X |  | X | X |
| 1. Han 2018 |  |  | X |  |  | X |  |
| 1. Lawlore 2004 |  | X |  |  |  |  |  |
| 1. Lawlore 2004 |  |  |  |  | X | X |  |
| 1. Pace 2020 | X |  | X |  |  | X |  |
| 1. Park 2017 |  |  | X | X |  | X | X |
| 1. Peng 2019 |  |  | X |  |  | X |  |
| 1. Perrin 2008 | X | X | X | X | X | X | X |
| 1. Powe 2017 | X |  | X | X |  | X | X |
| 1. Rollison B 2008 |  |  |  |  |  | X |  |
| 1. Rollison 2008 | X | X | X | X | X | X |  |
| 1. Sanderson (2010) |  |  | X |  |  | X |  |
| 1. Sella 2011 |  | X | X | X |  |  | X |
| 1. Troisi 1998 | X | X | X | X | X | X | X |
|  | 8 | 5 | 15 | 10 | 6 | 15 | 10 |
| CCA-37% |  |  |  |  |  |  |  |

# Table 5 Corrected covered area Matrix GDM and thyroid cancer

| Primary studies | Dong 2022 MA | Wang 2022 |
| --- | --- | --- |
| 1. Bejaimal 2016 | X | X |
| 1. Han 2018 | X | X |
| 1. Pace 2020 | X | X |
| 1. Peng 2019 | X | X |
|  | 4 | 4 |
|  |  |  |
| CCA=100% |  |  |

# Table 6 Corrected covered area Matrix GDM and pancreatic cancer

| Primary studies | Tong 2014 | Wang 2020 |
| --- | --- | --- |
| 1. Perrin 2008 | X | X |
| 1. Sella 2011 |  | X |
|  | 1 | 2 |
| CCA =50% |  |  |

# Table 7 Corrected covered area Matrix Pre-eclampsia and breast cancer

| Primary studies | KIM 2013 | Wang 2021 | Sun 2018 | Bellamy | Min Yao 2023 | Zohre 2018 |
| --- | --- | --- | --- | --- | --- | --- |
| 1. Bhattacharya 2012 |  | X | X |  | X |  |
| 1. Brasky 2013 |  |  |  |  |  | X |
| 1. Caldreon 2009 |  | X | X |  | X | X |
| 1. Cnattinigius 2005 |  |  | x |  |  |  |
| 1. Cohn 2001 |  |  | X | X | X | X |
| 1. Eckbom et al. 1992 [14] | X |  |  |  |  |  |
| 1. Ekbom et al. 1997 [23] | X |  |  |  |  |  |
| 1. Ekbom et al. 1997 [23] | X |  |  |  |  |  |
| 1. Innes et al. 2004 [18] | X |  |  |  |  | X |
| 1. Le Marchand et al. 1988 [15] | X |  |  |  |  |  |
| 1. Lin 2017 |  |  | X |  |  |  |
| 1. Ma 2010 |  |  | X |  | X | X |
| 1. Margalit et al. 2009 [22] | X |  |  |  |  |  |
| 1. Margalit et al. 2009 [22] | X |  |  |  |  |  |
| 1. Mogren 2001 |  | X | X | X | X |  |
| 1. Opdahl 2010 |  | X | X |  | X | X |
| 1. Pacheco 2015 |  | X | X |  | X | x |
| 1. Paltiel et al. 2004 [24] | X |  |  |  |  | X |
| 1. Paltiel et al. 2004 [24] | X |  |  | X |  |  |
| 1. Polendak et al. 1983 [16] | X |  |  |  |  |  |
| 1. Richardson et al. 2000 [19] | X |  |  |  |  | XX |
| 1. Terry et al. 2006 [17] | X |  |  |  |  | X |
| 1. Troisi et al. 1998 [20] | X |  |  |  |  | X |
| 1. Troisi et al. 2007, US [21] | X |  |  |  |  |  |
| 1. Troisi et al. 2007, US [21] | X |  |  |  |  |  |
| 1. Vatten et al. 2002 [26 | X |  |  |  |  |  |
| 1. Vatten et al. 2002 [26 | X |  |  |  |  | X |
| 1. Vatten et al. 2007 [25] | X | X |  |  |  | X |
| 1. Vatten et al. 2007 [25] | X | X |  | X |  |  |
| 1. Wallfisch 2015 |  | X | X |  | X |  |
| 1. Wright 2018 |  | X |  |  |  |  |
| 1. Yang a 2018 |  | X |  |  | X |  |
| 1. Yang b 2018 |  | X |  |  | X |  |
| 1. Serrand 2021 |  |  |  |  |  |  |
| 1. Nicholas 2023 |  |  |  |  | x |  |
|  | 19 | 11 | 10 | 4 | 11 | 14 |
| CC=16% |  |  |  |  |  |  |

# Table 8 Corrected covered area Matrix Pre-eclampsia and endometrial cancer

|  | Jordao 2023 | Wang F 2021 |
| --- | --- | --- |
| 1. Liu 2021 | X |  |
| 1. Trabet 2020 | X |  |
| 1. Cho 2019 | X | X |
| 1. Walfisch 2015 | X | X |
| 1. Bhattacharya 2012 | X | X |
| 1. Calderon Margalit 2009 | X | X |
| 1. Morgen 2001 | X |  |
|  | 7 | 4 |
| CCA=19% |  |  |

# Table 9 Corrected covered area Matrix Twin births and breast cancer

|  | Kim 2012 | Veisi 2023 | Zohre 2018 |
| --- | --- | --- | --- |
| 1. Polednak 1983 | X | X |  |
| 1. Olsen 1988 | X | X |  |
| 1. Innes 2004 | X | X | X |
| 1. Jacobson 1989 | X |  |  |
| 1. Nasca 1992 | X | X |  |
| 1. Heish 1993 | X | X |  |
| 1. Dietz 1995 | X | X |  |
| 1. La Vecchia 1996 | X | X |  |
| 1. Triosi 1998 | X | X | X |
| 1. Wyshak 1983 | X | X |  |
| 1. Lambe 1996 | X | X |  |
| 1. Murphy 1997 | X | X |  |
| 1. Albrektsen 1995 | X | X |  |
| 1. Wohlfahrt 1999 | X | X |  |
| 1. Neale 2004 | X | X | X |
| 1. Neale 2005 | X | X | X |
| 1. Jietal 2007 | X | X | X |
| 1. LM.Krul 2014 |  | X |  |
| 1. R Trois 2012 |  | x |  |
| 1. Lauren 2018 |  | x |  |
|  | 17 | 20 | 5 |
| CCA=85% |  |  |  |

# Table 10 Corrected covered area summary

| Index | Author,  Year | Quality | Pregnancy complication | Outcome | Data analysis | CCA | Decision |
| --- | --- | --- | --- | --- | --- | --- | --- |
| 1 | Flachs 2022 | Low | GDM | Breast Cancer | NA(8) | 42% | No |
|  | Hardefeldt  2012 | Low |  |  | MA(5) |  | No |
|  | Simon 2021 | Low |  |  | NA(15) |  | No |
|  | Xie 2019 | Moderate |  |  | MA(10) |  | No |
|  | Tong  2014 | Low |  |  | MA(6) |  | No |
|  | Wang  2020 | Moderate |  |  | MA(15) |  | Yes |
|  | Zohre 2018 | Low |  |  | MA(10) |  | No |
| 2 | Dong 2022 MA | Low | GDM | Thyroid cancer | MA(4) | 100% | No |
|  | Wang 2021 | Moderate |  |  | MA(4) |  | Yes |
| 3 | Tong 2014 | Low | GDM | Pancreatic cancer | MA(1) |  | No |
|  | Wang 2020 | Moderate |  |  | MA(2) |  | Yes |
| 4 | Kim 2013 | Low | Pre-eclampsia | Breast cancer | MA(19) |  | No |
|  | Wang 2021 | Moderate |  |  | MA(10) |  | No |
|  | Zohre 2018 | Low |  |  | MA(14) |  | No |
|  | Sun 2018 | Moderate |  |  | MA(11) |  | No |
|  | Bellamy 2007 | Moderate |  |  | MA(4) |  | No |
|  | Min Yao 2023 | Moderate |  |  | MA(11 |  | Yes |
| 5 | Wang F 2021 | Moderate | Pre-eclampsia | Endometrial cancer | MA (4) | 19% | No |
|  | Jardao 2023 | High |  |  | MA(7) |  | Yes |
| 6 | Kim 2012 | Low | Twin births | Breast cancer | MA (17) | 85% | No |
|  | Veisi 2023 | High |  |  | MA(20) |  | Yes |
|  | Zohre 2018 | Low |  |  | (MA5) |  | No |

# Table 11 Exposure definitions as used in the reviews

| Exposure | Definition | Reviews |
| --- | --- | --- |
| Miscarriage |  | Tong, H., et al.  Dick, M.L., et al.  Mannathazhathu, A.S., et al.  Zhong, G.C., et al. |
| Pre-eclampsia | New onset of hypertension (over 140 mmHg systolic or over 90 mmHg diastolic) after 20 weeks of pregnancy and the coexistence of 1 or more of the following new-onset conditions: - proteinuria - other maternal organ dysfunction (renal insufficiency, liver involvement, neurological complications, haematological complications) - uteroplacental dysfunction | Jordao, H., et al.  Wang, F., et al.  Bellamy, L., et al |
| Pre-eclampsia | New hypertension presenting after 20 weeks of pregnancy without significant proteinuria and | Yao, M., et al. |
| Molar pregnancy | This review defined the molar pregnancy as abnormal pregnancy, either complete (no fetal tissue) or partial (abnormal fetal tissue), diagnosed by ultrasound and/or histological or genetic confirmation. | Xie, C., et al. |
| Gestational diabetes Mellitus | hyperglycaemia in pregnancy that is above diagnostic thresholds for diabetes  a fasting plasma glucose level of 5.6 mmol/litre or above or  a 2-hour plasma glucose level of 7.8 mmol/litre or above | Wang, Y., et al. |
| Preterm birth | Babies born alive before 37 weeks of pregnancy are completed. | Veisi, P., et al. |
| Caesarean birth | Birth of a singleton/ multiple baby/babies baby between 37+0 and 42+6 weeks of gestation, by caesarean section(elective or emergency | Douligeris, A., et al |
| Multiple birth | Term used when one is expecting two or more babies at the same time (twins, triplets or more) | Razavi, M., et al. |
